# Validation of a Low-Cost Paper-Based Screening Test for Sickle Cell Anemia

**DOI:** 10.1371/journal.pone.0144901

**Published:** 2016-01-06

**Authors:** Nathaniel Z. Piety, Xiaoxi Yang, Julie Kanter, Seth M. Vignes, Alex George, Sergey S. Shevkoplyas

**Affiliations:** 1 Department of Biomedical Engineering, Tulane University, New Orleans, Louisiana, United States of America; 2 Sickle Cell Center of Southern Louisiana, Tulane University School of Medicine, New Orleans, Louisiana, United States of America; 3 Department of Pediatrics, Section of Hematology/Oncology, Tulane University School of Medicine, New Orleans, Louisiana, United States of America; 4 Department of Pediatrics, Section of Hematology-Oncology, Baylor College of Medicine, Houston, Texas, United States of America; Université Claude Bernard Lyon 1, FRANCE

## Abstract

**Background:**

The high childhood mortality and life-long complications associated with sickle cell anemia (SCA) in developing countries could be significantly reduced with effective prophylaxis and education if SCA is diagnosed early in life. However, conventional laboratory methods used for diagnosing SCA remain prohibitively expensive and impractical in this setting. This study describes the clinical validation of a low-cost paper-based test for SCA that can accurately identify sickle trait carriers (HbAS) and individuals with SCA (HbSS) among adults and children over 1 year of age.

**Methods and Findings:**

In a population of healthy volunteers and SCA patients in the United States (n = 55) the test identified individuals whose blood contained any HbS (HbAS and HbSS) with 100% sensitivity and 100% specificity for both visual evaluation and automated analysis, and detected SCA (HbSS) with 93% sensitivity and 94% specificity for visual evaluation and 100% sensitivity and 97% specificity for automated analysis. In a population of post-partum women (with a previously unknown SCA status) at a primary obstetric hospital in Cabinda, Angola (n = 226) the test identified sickle cell trait carriers with 94% sensitivity and 97% specificity using visual evaluation (none of the women had SCA). Notably, our test permits instrument- and electricity-free visual diagnostics, requires minimal training to be performed, can be completed within 30 minutes, and costs about $0.07 in test-specific consumable materials.

**Conclusions:**

Our results validate the paper-based SCA test as a useful low-cost tool for screening adults and children for sickle trait and disease and demonstrate its practicality in resource-limited clinical settings.

## Introduction

Sickle cell anemia (SCA) is the most severe and prevalent form of sickle cell disease, accounting for more than 80% of all affected births worldwide.[1, 2] SCA results from homozygous inheritance of a missense mutation that induces a single amino acid change in the β-globin subunit of adult hemoglobin, converting normal adult hemoglobin (HbA) into sickle hemoglobin (HbS). Other less common forms of sickle cell disease occur when HbS is co-inherited with hemoglobin C (HbSC disease), β-thalassemia (HbSβ-thalassemia), or other abnormal hemoglobins. As a result of the mutation, the molecules of HbS gain the ability to polymerize into long fibers under hypoxic conditions, inducing a number of structural and functional abnormalities in affected erythrocytes. The cumulative effect of these abnormalities is an increased propensity of sickle erythrocytes to undergo intravascular lysis or trigger episodic occlusion of blood vessels. These abnormalities in turn result in systemic ischemia-reperfusion injury, chronic inflammation, activation of the coagulation system, and vascular dysfunction that induce the various clinical manifestations of SCA, giving rise to life-long morbidity and premature mortality associated with the disease.[3]

Children under 5 years of age are at increased risk of death from infections and other life-threatening complications of SCA, most of which can be prevented through highly effective and inexpensive prophylaxis (e.g. prophylactic administration of penicillin, pneumococcal immunizations, distribution of malaria bed nets, and education of parents about the importance of seeking medical attention for fever) if SCA is diagnosed early in life.[4, 5] SCA is normally diagnosed by measuring the HbS content (%HbS) in blood of patients with high-performance liquid chromatography (HPLC)[6], hemoglobin electrophoresis (HE)[7] or isoelectric focusing (IEF)[8]. Universal screening of newborns in the United States and many other developed countries using these highly accurate laboratory methods has enabled early intervention and treatment of SCA, which have significantly reduced SCA-related early childhood mortality and have contributed to the overall improvement in the quality of life and life-span of adults with SCA.[9, 10]

Although prevalent worldwide due to population migration, SCA is most common in sub-Saharan Africa where more than 200,000 affected births occur every year. Recent data from a newborn screening program in Angola, for example, indicates incidence at birth of approximately 21% for sickle trait (HbAS) and approximately 1.5% for SCA (HbSS).[11, 12] Estimates suggest that as many as 50–90% of the children born with SCA in low-income developing countries of sub-Saharan Africa will die before the age of 5.[13, 14] Universal newborn screening using existing technologies may not be currently feasible in these settings because of the large number of out-of-hospital births,[15] prohibitively high cost of laboratory methods needed for screening newborns, and logistical constraints associated with contacting parents of affected infants for counseling and preventive care after postnatal discharge. For similar reasons, there have been few population-wide estimates of the prevalence of SCA or attempts to screen for carriers of sickle trait for purposes of genetic counseling. An alternative approach to identifying and establishing care for SCA patients would be to screen older infants at routine postnatal medical visits, or via large-scale outreach efforts since major complications are rare in the first six months of infancy.[16] The development and validation of a simple, rapid, and inexpensive screening test for SCA would represent a significant advance that could transform clinical care for this disease in low-income developing countries.

Our research laboratory has recently developed an easy-to-use, inexpensive, rapid test for SCA that could accurately identify normal individuals (HbAA), sickle trait carriers (HbAS) and individuals suffering from SCA (HbSS) in a population of adults and children over one year of age. Our test operates via visual interpretation of the blood stain patterns produced on chromatography paper by a drop of blood mixed with a hemoglobin (Hb) solubility buffer due to the difference in transport of insoluble sickle hemoglobin (HbS) and soluble forms of hemoglobin (HbA, HbF, HbC) through the paper substrate.[17–19] In this manuscript, we describe further development of the paper-based test and its initial clinical validation for identifying individuals with sickle cell trait (HbAS), sickle cell anemia (HbSS) and other forms of sickle cell disease (HbSC, HbSβ- thalassemia) in our research laboratory in the United States. Furthermore, we demonstrate the utility of the paper-based test in resource-limited settings by evaluating its diagnostic accuracy for screening post-partum women with previously unknown SCA status for sickle cell trait or disease in a clinical laboratory of the sickle cell screening program in Cabinda, Angola.

## Materials and Methods

### Blood samples (New Orleans, Louisiana, United States)

Venous blood samples were obtained with written informed consent in 4mL Vacutainer tubes (K_2_EDTA, BD, Franklin Lakes, NJ) from healthy volunteers and patients of the Pediatric Hematology- Oncology Clinic (Tulane University Hospital) and the Sickle Cell Center of Southern Louisiana (New Orleans, LA). Samples from patients who received blood transfusions in the previous three months were excluded. Blood samples were kept at room temperature (20–25°C) following collection by venipuncture and used within 4–5 hours after venipuncture. The hemoglobin content of blood samples from sickle cell trait carriers and sickle cell disease patients was quantified via hemoglobin electrophoresis at the Children’s Hospital of Oakland Research Laboratory (Oakland, CA). A total of 55 patients were included in the United States study population. The study protocol was approved by Tulane University Biomedical Institutional Review Board.

### Blood samples (Cabinda, Angola)

Blood samples were obtained with written informed consent from post-partum mothers of unknown sickle cell status at the Primero de Maio obstetric hospital (Cabinda, Angola). Capillary blood samples were collected by finger-stick into K_2_EDTA coated collection tubes (Microvette, Sarstedt AG & Co., Germany) and onto paper cards (Whatman™ 903 Neonatal Screening Protein Saver Cards, GE Healthcare, USA). Subsequent testing on these samples was performed at the newborn screening laboratory of the Clinica de Celulas Falciformes at the Dispensario Materno Infantil in Cabinda. Blood spots on paper cards were allowed to dry completely before being used for isoelectric focusing (IEF) electrophoresis to qualitatively determine the presence of hemoglobin variants. Liquid blood samples were refrigerated following collection and used within 21 days after collection for the rapid SCA diagnostic test. A total of 226 patients were included in the Angola study population. This part of research was approved by the Ethics Committee of the Scientific Council of the Faculty of Medicine, Universidad 11 de Noviembre (Cabinda, Angola), and by the Institutional Review Board for Human Subject Research for Baylor College of Medicine and Affiliated Hospitals.

### Hemoglobin solubility buffer

The hemoglobin solubility buffer used in this study consisted of three components: saponin, sodium hydrosulfite and a concentrated phosphate buffer.[17, 18] Potassium phosphate buffer at 2.49M was made by dissolving solid 1.24M (169 g/L) monobasic and 1.25M (217 g/L) dibasic potassium phosphate in deionized water (final concentration of 2.49M). Saponin (4g/L) irreversibly lyses red blood cells (RBCs) by creating holes in the lipid bilayer, thereby releasing hemoglobin into the buffer.[20] Sodium hydrosulfite (30 g/L) then converts the released hemoglobin into deoxy-Hb that is either soluble (HbA, HbE, HbF or HbC) or insoluble (HbS) in the phosphate buffer.[21, 22] Saponin and sodium hydrosulfite were stored as dry reagents and concentrated phosphate buffer was stored as an aqueous solution. Hemoglobin solubility buffer was reconstituted at the site where the tests were performed, stored at ambient temperature and used within 1 day. For additional information on optimization of the Hb solubility buffer and stability of the assay please see **S1 and S2 Figs**.

### Automated scoring of the blood stain patterns

The sheets of paper containing an array of dried blood stains were digitized with a portable flatbed scanner (CanoScan LiDE110, Canon USA Inc, Lake Success, NY) and the scanned images were analyzed with a custom image analysis algorithm (MATLAB, The Math Works Inc, Natick, MA). The quantitative blood stain pattern analysis was based on the RGB values of the digitized images. The color data from the Blue (B) channel of each pixel was used to quantify the red color intensity of multiple sections of the blood stain (<color intensity> = <255 –B>). S-index was defined as the quotient of mean red color intensity of pixels in the center area of the blood stain and mean color intensity of pixels in the ring area of the blood stain. The S-index represents the ratio of HbS (insoluble when deoxygenated in concentrated phosphate buffer) to other (soluble) forms of hemoglobin present in the sample. C-index was defined as the product of the total red color intensity of the dark red center spot and the average red color intensity at a distance of 5 mm from the center of the blood stain. The C-index represents the amount of HbS and the total amount of Hb present in the sample.

### Evaluation of test performance

Sensitivity = TP / (TP + TN); specificity = TN / (FP + TN); positive predictive value (PPV) = TP / (TP + FP); negative predictive value (NPV) = TN / (TN + FN); and accuracy = (TP + TN) / (TP + FP + TN + FN) were calculated; where TP = true positive, FP = false positive, TN = true negative and FN = false negative. 95% confidence intervals were calculated using the Wilson method.[23] Receiver operating characteristic (ROC) curves were created using a custom algorithm (MATLAB, The Math Works Inc, Natick, MA). Fleiss’ kappa was calculated to determine inter-operator agreement for visual diagnoses.[24]

## Results

### Visual diagnosis of sickle cell trait and sickle cell anemia with the paper-based test

**Fig 1:**
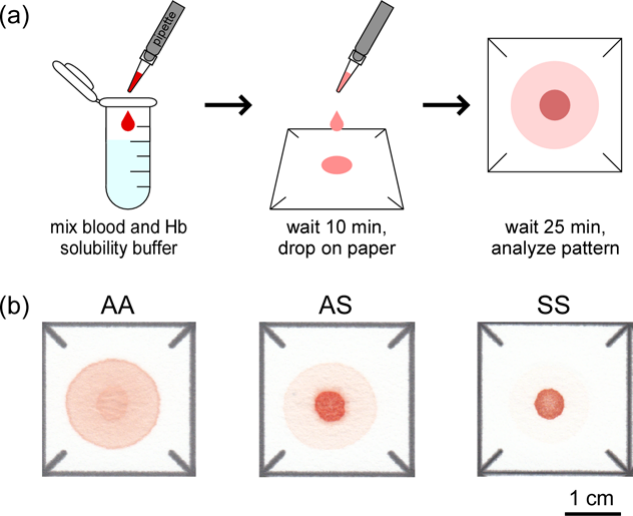
Overview of the paper-based SCA diagnostic test. (**a**) To perform the test, a 20 μL droplet of blood mixed 1:10 (by volume) with Hb solubility buffer–a concentrated phosphate buffer (2.49M) containing a hemolytic (saponin) and a reducing agent (sodium hydrosulfite)–was deposited on paper. Differential transport of polymerized HbS and soluble forms of Hb in the paper substrate produced blood stains with characteristic patterns. (**b**) Representative images of the blood stain patterns produced by HbAA, HbAS and HbSS samples.

We described the design and operation of the paper-based SCA test previously.[17, 18] Briefly, to perform the test a sample of blood is diluted with the hemoglobin solubility buffer (see **S1 Methods**) at a 1:10 ratio (by volume) and then a 20 μL droplet of this mixture is deposited onto chromatography paper (**Fig 1A**). The insoluble polymerized deoxy-HbS and cellular debris are entangled by the paper fibers, remaining within the original outline of the droplet deposited on paper while soluble forms of hemoglobin are transported laterally outwards by capillary action. This process produces a blood stain with an easily recognizable pattern of red color intensity representative of the Hb content of the blood sample (**Fig 1B**).[18]

**Fig 2:**
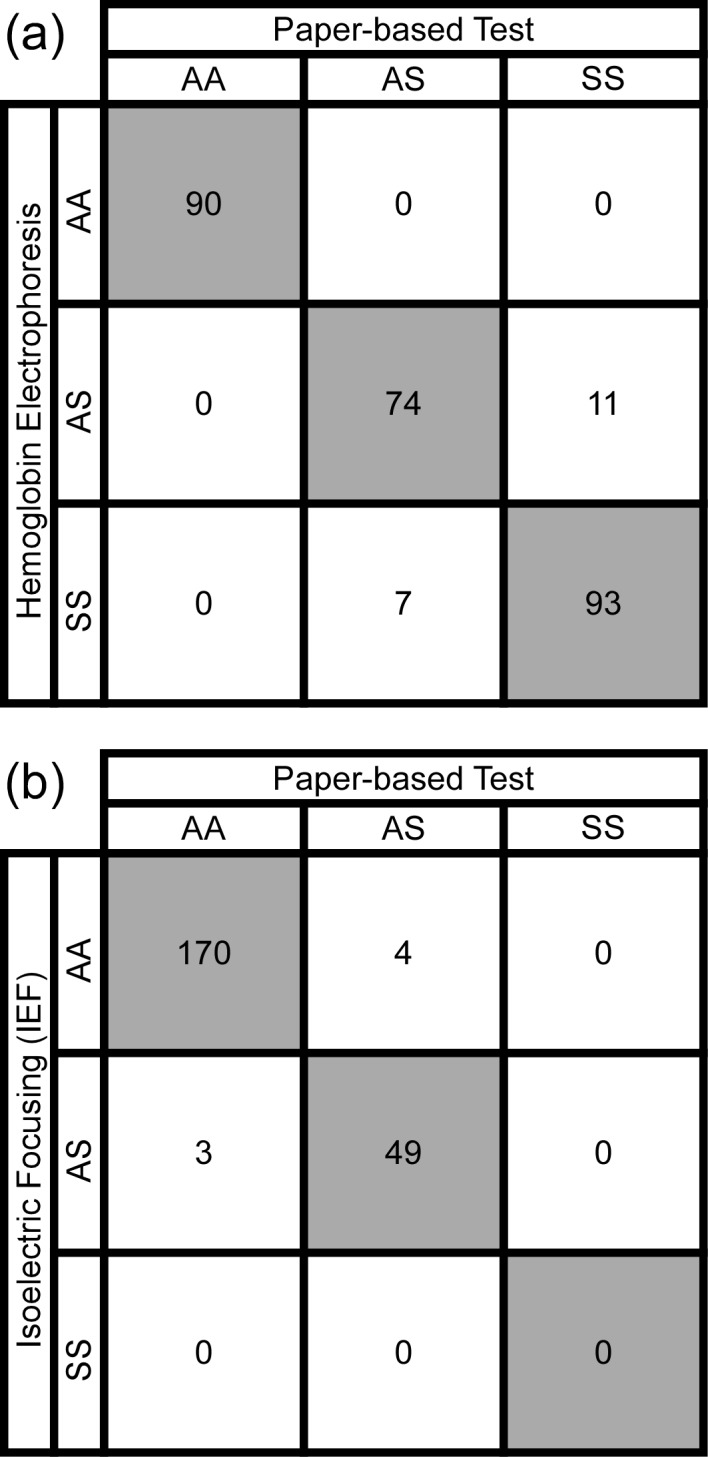
Validation of the paper-based SCA test in a research laboratory and in a resource-limited clinical setting (Cabinda, Angola). (**a**) Aggregate confusion matrix for the diagnoses of blood samples collected from normal volunteers and patients of Pediatric Hematology-Oncology Clinic at Tulane University Hospital and of the Sickle Cell Center of Southern Louisiana (New Orleans, LA) performed via visual evaluation of the blood stains by human scorers (n = 5). Rows correspond to true genotypes (diagnosed by hemoglobin electrophoresis) and columns correspond to predicted genotypes (diagnosed by the paper-based test). Shaded cells along the diagonal contain numbers of correct diagnoses. (**b**) Confusion matrix for the diagnoses of blood samples collected at the Primero de Maio obstetric hospital from postnatal females with unknown SCA status. The rapid test was performed and interpreted via visual evaluation by healthcare workers at the newborn screening laboratory of the Clinica de Celulas Falciformes at the Dispensario Materno Infantil (Cabinda, Angola). Rows correspond to true genotypes (diagnosed by isoelectric focusing) and columns correspond to predicted genotypes (diagnosed by the paper-based test). Shaded cells along the diagonal contain numbers of correct diagnoses.

We hypothesized that the characteristic differences between the patterns of blood stains produced by samples from individuals with normal Hb expression (HbAA), sickle cell trait carriers (HbAS) and sickle cell anemia patients (HbSS) (**Fig 1B**) would permit conclusive identification of subjects of each type by visual classification. We tested this hypothesis using venous blood samples from healthy volunteers and from patients and parents of the Pediatric Hematology-Oncology Clinic (Tulane University Hospital) and the Sickle Cell Center of Southern Louisiana (New Orleans, LA) under an approved IRB protocol. Our study population included children age 12 months and older and adults (n = 55), with hemoglobin concentrations ranging from 6.2 to 16.5 g/dL, HbS levels ranging from 0 to 93% and HbF levels ranging from 0 to 28%. Five novice users independently scored blood stains (presented in a randomized order) from HbAA (n = 18), HbAS (n = 17) and HbSS (n = 20) blood samples as HbAA, HbAS or HbSS. **Fig 2A** shows the aggregate confusion matrix of the visual diagnoses made by all scorers. The paper-based SCA test was able to detect the presence of any HbS in a sample (i.e., distinguish HbAS and HbSS from HbAA) with mean sensitivity of 100% (SD, standard deviation = 0%) and specificity of 100% (SD = 0.0%). The test was able to identify HbSS samples (i.e., distinguish HbSS from HbAS and HbAA) with sensitivity of 93.0% (SD = 7.5%) and specificity of 93.7% (SD = 2.8%). The test was able to distinguish HbSS samples from HbAS samples with sensitivity of 93.0% (SD = 7.5%) and specificity of 87.1% (SD = 5.8%). **Table 1**contains a summary of the mean and standard deviation of other performance metrics for diagnoses made via visual evaluation of the rapid paper-based test. The Fleiss’ kappa calculated for all scored images was 0.91, suggesting an almost perfect agreement between the five scorers in reading the presented test stains.

**Table 1:** Visual diagnosis performance metrics.

	AA vs (AS and SS)	SS vs (AA and AS)	AS vs SS
**Sensitivity**	100.0 ± 0.0%	93.0 ± 7.5%	93.0 ± 7.5%
**Specificity**	100.0 ± 0.0%	93.7 ± 2.8%	87.1 ± 5.8%
**PPV**	100.0 ± 0.0%	89.8 ± 3.8%	89.8 ± 3.8%
**NPV**	100.0 ± 0.0%	96.2 ± 3.9%	92.4 ± 7.5%
**Accuracy**	100.0 ± 0.0%	93.5 ± 1.5%	90.3 ± 2.2%

Summary of performance metrics for visual diagnoses made by different scorers (n = 5) on blood samples obtained from healthy subjects and SCD patients in New Orleans, LA. Mean values ± SD.

### Validation of the paper-based SCA test in a resource-limited setting

The sickle cell newborn screening program in Cabinda, Angola currently collects blood samples from neonates born at the Primero de Maio obstetric hospital for early diagnosis by isoelectric focusing (IEF) gel electrophoresis. We reasoned that the mothers of these babies presented an easily accessible population in which to assess the feasibility and diagnostic accuracy of our paper-based SCA screening test, particularly since we could concurrently perform IEF analysis for determination of their exact sickle cell status.

Two healthcare workers at the Cabinda sickle cell newborn screening laboratory (who had no prior experience with the test) were trained to collect samples of capillary blood from these subjects via finger prick, process them using the paper-based test, and score them visually as either HbAA, HbAS, or HbSS. A total of 276 subjects were enrolled on the study from the 400 eligible mothers seen at the hospital during this recruitment phase, and 226 samples were successfully processed and independently scored with both the paper-based SCA test and IEF electrophoresis (**S5 Fig**). **Fig 2B** shows the confusion matrix of the visual diagnoses made by the health workers. The paper-based SCA test was able to detect the presence of any HbS in the sample (i.e., distinguish HbAS and HbSS from HbAA subjects) with sensitivity of 94.2% (95% confidence interval, CI: 90.4–96.6%), specificity of 97.7% (CI: 94.8–99.0%), positive predictive value of 92.5% (CI: 88.3–95.2%), negative predictive value of 98.3% (CI: 95.6–99.3%) and overall diagnostic accuracy of 96.9% (CI: 93.7–98.5%). Additionally, we found that none of the post-partum women in our study had HbSS, likely due to our relatively small sample size, the decreased prevalence of SCA in the adult population due to early mortality, and the low likelihood of a woman with SCA carrying a baby to term without intensive prenatal care in that setting.

### Automated diagnostics of sickle cell trait and sickle cell anemia with the paper-based test

**Fig 3:**
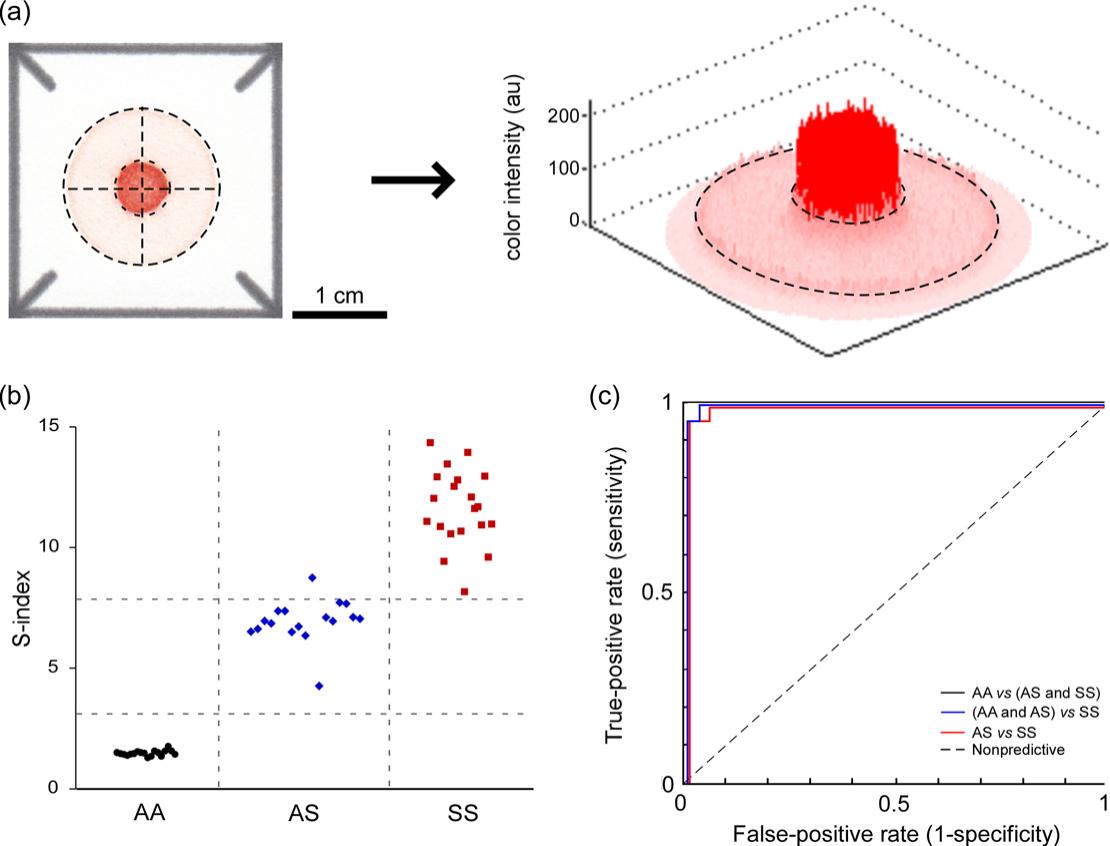
Automated analysis of blood stains in paper. (**a**) A custom image analysis algorithm automatically detected the center of each blood stain (dashed crosshair) and extracted the RGB values for all pixels contained within the dark red center spot (smaller dashed circle) and within the pink peripheral ring (area between the smaller and the larger dashed circles). The S-index was defined as the quotient of the mean red color intensity of the center spot and that of the peripheral ring of the blood stain. (**b**) The values of the S-index for (●) HbAA (n = 18), (♦) HbAS (n = 17) and (■) HbSS (n = 20) samples obtained from healthy subjects and patients in New Orleans, LA. (**c**) Receiver operating characteristic (ROC) curves for the use of S-index to identify HbAA, HbAS and HbSS samples. The area under the curve (AUC) for discriminating HbAA from HbAS and HbSS was 1.00, the AUC for discriminating HbSS from HbAA and HbAS was 0.9986 and the AUC for discriminating HbSS from HbAS was 0.9971.

Optionally, once the newly formed blood stain dries out completely (<25 minutes at 18–22°C room temperature and 20–80% humidity), the sheet of chromatography paper containing an array of such blood stains can be digitized using a portable flatbed scanner.[17, 18] To permit quantitative and automated scoring of samples, we developed a custom image analysis algorithm (implemented in MATLAB) to calculate an S-index from the digitized blood stains. We defined S-index as the quotient of mean red color intensity of pixels in the center area of the blood stain and mean color intensity of pixels in the ring area of the blood stain (**Fig 3A**). Because the mean color intensity of the center spot is proportional to the amount of HbS (rendered insoluble by the hemoglobin solubility buffer), and the mean color intensity of the peripheral ring is proportional to the amount of soluble forms of hemoglobin (HbA, HbF and HbC), the S-index represents the fraction (or percentage) of HbS in the total hemoglobin content of the sample. The concentration of hemoglobin (inclusive of all hemoglobin variants) in the sample droplet determines the overall brightness of the blood stain produced on paper, and may vary significantly due to variation of blood hemoglobin among subjects and imperfections of the sample preparation protocol. Although variations in hemoglobin concentration change the absolute values of the mean color intensity of the center spot and the peripheral ring, they do not affect the value of S-index, which is calculated as a quotient of the two color intensities.[17, 18]

**Fig 3**demonstrates the ability of the S-index to differentiate between HbAA, HbAS and HbSS blood samples in the same cohort in the United States used for the visual scoring described above. Using the S-index, our paper-based SCA test was able to detect the presence of any HbS in the sample (i.e., distinguish HbAS and HbSS from HbAA) with sensitivity of 100% (CI: 93.5–100.0%) and specificity of 100% (CI: 93.5–100.0%) (**Fig 3B**). The test was able to identify HbSS samples (i.e., distinguish HbSS from HbAS and HbAA) with sensitivity of 100% (CI: 93.5–100.0%) and specificity of 97.1% (CI: 88.8–99.3%) (**Fig 3B**). The test was able to distinguish HbSS samples from HbAS samples with sensitivity of 100% (CI: 90.6–100.0%) and specificity of 94.1% (CI: 81.6–98.3%) (**Fig 3B**). **Fig 3C** shows the receiver operating characteristic (ROC) curves for the use of S-index to identify HbAA, HbAS and HbSS samples. Based on the data from n = 55 subjects, the area under the curve (AUC) for discriminating HbAA from HbAS and HbSS was 1.00, the AUC for discriminating HbSS from HbAA and HbAS was 0.9986 and the AUC for discriminating HbSS from HbAS was 0.9971 (**Fig 3C**). **Table 2**contains a summary of the mean and standard deviation of other performance metrics for diagnoses made via automated evaluation of the rapid paper-based test.

**Table 2:** Automated diagnosis performance metrics.

	AA vs (AS and SS)	SS vs (AA and AS)	AS vs SS
**Sensitivity**	100%	100%	100%
**Specificity**	100%	97%	94%
**PPV**	100%	95%	95%
**NPV**	100%	100%	100%
**Accuracy**	100%	98%	97%

Summary of performance metrics for automated diagnoses made by the image analysis algorithm on blood samples obtained from healthy subjects and SCD patients in New Orleans, LA.

### Using the paper-based SCA test for diagnosing other forms of sickle cell disease

**Fig 4:**
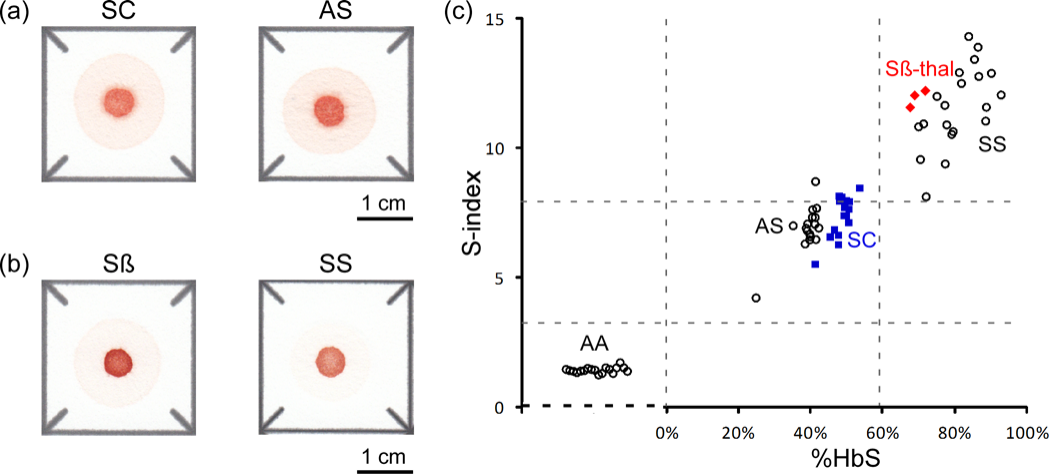
Diagnosis of other forms of sickle cell disease. (**a**) Representative images of blood stains produced in paper by HbSC samples and by HbAS samples, for comparison. (**b**) Representative images of blood stains produced in paper by HbSβ^+^-thalassemia samples and by HbSS samples, for comparison. (**c**) Classification of HbSC and HbSβ^+^-thalassemia samples in the S-index domain. The values of the S-index for (○) HbAA, HbAS and HbSS samples (n = 55), (■) HbSC (n = 16) and (♦) HbSβ^+^-thalassemia (n = 3) are shown on Y-axis. The location along the X-axis of each data point for HbAS, HbSS, HbSC and HbSβ^+^-thalassemia samples corresponds to their %HbS measured with Hb electrophoresis by an off-site clinical laboratory. HbAA samples (n = 18) contained no HbS; hence the random lateral spread of the data points representing these samples on the plot.

The easily distinguishable patterns of blood stains produced by HbAA, HbAS, and HbSS samples on paper enabled direct and relatively straightforward diagnostics (**Fig 1B**). We could not, however, visually distinguish between HbAS and HbSC samples (**Fig 4A**) or between HbSS and HbSβ^+^-thalassemia samples (**Fig 4B**). Similarly, in automated analysis based on S-index HbSβ^+^-thalassemia samples (n = 3) were indistinguishable from samples in the HbSS group because of the high HbS content in both sets of samples (> 60% HbS for HbSβ^+^-thalassemia) (**Fig 4C**). Likewise, HbSC samples (n = 16) grouped very closely with HbAS samples because of the relatively small difference between their HbS content (38–42% for HbAS and 41–50% for HbSC subjects in this study) (**Fig 4C**).

**Fig 5:**
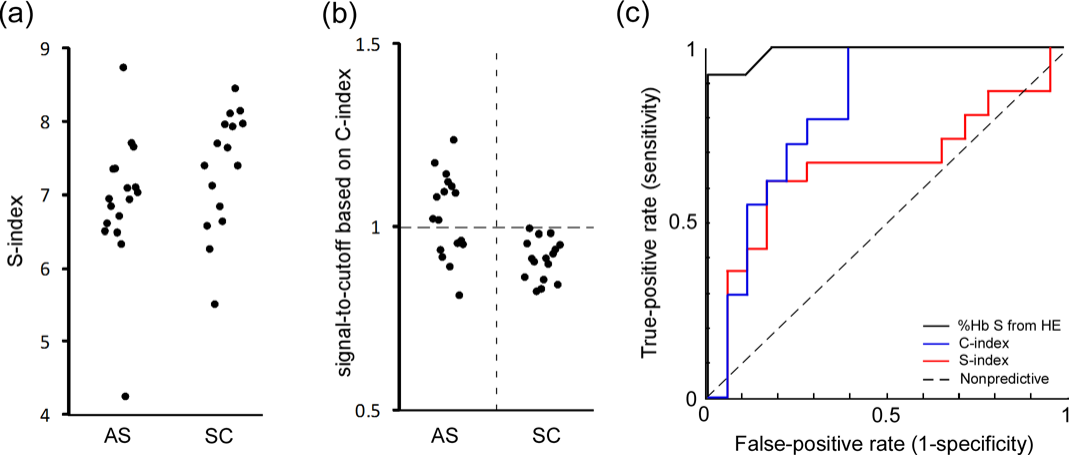
Differentiation of HbSC samples from HbAS samples via automated image analysis. (**a**) The values of S-index for HbAS (n = 17) and HbSC (n = 16) samples. (**b**) Vertical scatter plots of C-index (normalized by cutoff values) for HbAS and HbSC samples. C-index was defined as the product of total color intensity of all pixels in the center area of the blood stain and average color intensity of all pixels at a distance of 5 mm from the center of the blood stain. (**c**) ROC curves for differentiating HbSC and HbAS based on (-) S-index, AUC = 0.67; (-) C-index, AUC = 0.82; and (-) %HbS by electrophoresis, AUC = 0.99.

To address this limitation (the inability of the paper-based test to accurately differentiate HbAS and HbSC samples) we devised an alternative metric dubbed C-index, which was defined as the product of total red color intensity of all pixels in the center area of the blood stain and average red color intensity of all pixels at a distance of 5 mm from the center of the blood stain. The C-index takes into account the absolute concentration of Hb present in the sample, unlike the S-index which is based on only the relative concentrations of HbS and other forms of Hb. The inclusion of absolute Hb concentration in the calculation of the C-index enables it to distinguish between HbSC and HbAS because HbSC samples typically have lower Hb concentrations that HbAS samples. The C-index was able to differentiate HbSC from HbAS samples (**Fig 5**) with sensitivity of 100% (CI: 89.6–100.0%) and specificity of 59.0% (CI: 42.2–74.0%). In the ROC analysis, the AUC for the use of S-index to differentiate HbSC and HbAS was 0.67, and the AUC for the use of C-index was 0.82 (and the AUC for the use of %HbS data measured by Hb electrophoresis to distinguish HbSC from HbAS was 0.99).

## Discussion

Population-wide screening for SCA using conventional diagnostic methods (e.g., HE, IEF or HPLC) is currently impractical because of the prohibitively high cost and lack of access to the technical infrastructure required for such testing. A more feasible approach, given the limited resources available for healthcare in low-income developing countries and the relatively low incidence of the disease in the general population (e.g., ~21% sickle cell trait and ~1.5% sickle cell anemia at birth in Angola),[11, 12] could be to use a low-cost screening assay with a low false-negative rate for general population screening, and then perform high-cost laboratory testing to confirm the diagnosis only for ambiguous cases.

In this paper, we present the initial clinical validation of the paper-based SCA assay we engineered previously to enable such an approach. Our assay, which relies on the differential wicking of insoluble sickle Hb and soluble non-sickle Hb in paper, is capable of distinguishing HbSS and HbAS samples from each other and from samples free of sickle Hb with a high degree of accuracy and reproducibility. Our test can identify individuals whose blood contains any HbS with 94.2% sensitivity and 97.7% specificity via visual classification of the blood stains (and with 100% sensitivity and 100% specificity via automated analysis). Such diagnostic accuracy approaches that of much more technologically complex methods.

Our paper-based SCA test also offers a number of advantages that are highly relevant in resource-limited settings. It is simple and rapid to use, requiring only three steps for completion and permitting reliable visual scoring of results within 30–40 minutes of sample collection. This simplicity also makes the test portable for use in outlying facilities where standard laboratory equipment may not be available and distance, turn-around time, and poor communication infrastructure can limit the usefulness of screening outreach.

The cost of test-specific reagents and materials for the paper-based test is $0.07 per test (**S1 Table**). The cost of materials necessary to collect and meter blood (typically not packaged with commercially available test kits or included in per-test cost estimates) is about $0.70. The total per-test cost for all components necessary to perform the paper-based test is therefore $0.77 (for a detailed cost breakdown see **S1 Table**). Samples can be processed individually or in batched lots without significantly affecting the per-test costs. The cost of optional equipment (i.e., laptop and portable scanner) to perform automated analysis of blood stains is less than $400. In contrast, the estimated per-test cost for conventional IEF and HPLC at our collaborating clinical center in the United States is approximately $60, and the estimated cost per sample for IEF testing in the recently established newborn screening program in Angola is $4.94 when samples are batched in large lots.[11, 18] Additionally, these conventional methods require specialized equipment and trained technicians, representing large initial investments for each new testing site. As such, the rapid test offers significant cost saving advantages compared to other methods of SCA diagnosis.

The paper-based test also has the virtue of being relatively insensitive to variations in sample storage, lysate preparation, and stain development and storage (see **S3 and S4 Figs**). Although we did not systematically test the effect of temperature and humidity on performance of the test, during this study the temperature in our research laboratory in the United States and in the clinical laboratory in Cabinda, Angola varied from 20 to 30°C and humidity varied from 20 to 90% without a noticeable effect on the assay performance. These observations suggest that our paper-based SCA assay is reasonably stable with respect to various environmental challenges. Finally our results obtained in Cabinda indicate that the paper-based assay is practical and feasible in a resource-limited setting and can be performed and interpreted by healthcare workers in such settings after only a few hours of informal training.

Our paper-based SCA test represents a significant improvement over existing assays based on Hb solubility (e.g., SickleDex^TM^). These assays allow the operator to visually confirm the presence of HbS within a sample, but, unlike our paper-based test, cannot distinguish between sickle trait and disease. Various modifications of the standard hemoglobin solubility assay that enable differentiation of sickle trait from disease rely on complex sample preparation procedures involving additional laboratory equipment and analytical instruments for detection,[25–27] and therefore have limited applicability in resource-limited settings of low-income developing countries.

Tests based on sickling of intact RBCs require relatively complex and expensive equipment such as a conventional microscope or a smartphone equipped with a microscope attachment to make the observation, and even then have difficulty to accurately distinguish sickle trait from disease because both types of samples contain at least some sickled cells. Recent advances in the field of microfluidics have produced an array of highly innovative research tools that can distinguish flow patterns of sickle and non-sickle blood samples.[28] These microfluidic devices, however, are largely impractical as low-cost diagnostic tools because of their complexity, very high cost, need for highly skilled personnel and a heavy reliance on expensive laboratory equipment for inducing RBC sickling, device operation and performing the measurements.

The presence of a small, yet significant fraction of dense RBCs (a result of loss of K^+^ content and cellular dehydration) is another hallmark of SCA[29] that has been used as the basis for a potentially low-cost diagnostic method (dubbed SCD-AMPS).[30, 31] Unlike our paper-based test, the SCD-AMPS test is not equipment- and electricity-free because it requires centrifugation of the blood sample placed on a column of density gradient medium to isolate the small fraction of dense RBCs and render the diagnosis.[30] The most current published version of the SCD-AMPS test kit includes specially modified capillaries containing the density gradient medium, a centrifuge and a car battery needed to power the centrifuge,[31] although slower, manual centrifuges could likely be used instead of a conventional microcentrifuge.[32] A recent field evaluation of the SCD-AMPS test in a large case-control study conducted in Zambia revealed that the diagnostic accuracy of the test was significantly diminished by high levels of HbF, clotting of the blood sample, variation between batches of the specially modified capillaries and the density gradient medium, and the environmental conditions during shipping of the special capillaries. Unlike our paper-based test, the SCD-AMPS test could not differentiate between normal individuals (HbAA) and sickle trait carriers (HbAS). The SCD-AMPS test was able to identify SCA patients (HbSS) with a sensitivity of 86% (82–90%) and a specificity of only 60% (53–67%),[31] demonstrating diagnostic accuracy that is substantially lower than our paper-based test. Although earlier reports suggested that SCD-AMPS test could also differentiate between SCA (HbSS) and HbSC disease, unfortunately none of the patients in the Zambia study had HbSC and therefore it was not possible substantiate this important capability in the field.[31]

RBCs containing HbS are known to undergo hemolysis when deoxygenated in isotonic solutions of certain non-electrolytes because of the changes in RBC membrane caused by deoxy-HbS polymerization.[33] A diagnostic test based on monitoring RBC hemolysis in non-electrolyte solutions has been proposed for diagnosing SCA.[34] However, complicated sample preparation, lengthy incubation time, the need for precise control of pH and gasses, and the specialized equipment needed to interpret the results (by quantifying the change in optical density), could make this test difficult to use in a resource-limited setting.

Finally, a conventional lateral flow immunoassay for diagnosing SCD at the point-of-care has recently been developed (dubbed Sickle SCAN™).[35] This test uses proprietary antibodies to identify the presence of HbA, HbS and HbC within a whole blood sample, and thereby diagnose HbAA, HbAS, HbSS, HbSC and HbSβ^+^-thalassemia. Initial evaluation of the test in a controlled environment of a clinical laboratory and in a clinic in the U.S. has demonstrated excellent diagnostic accuracy in a population of adults and children older than 5 weeks of age.[35] According to the Sickle SCAN™ product insert, the test must be stored and used within a relatively narrow range of temperature (2–30°C), which may preclude the use of this test in resource-limited settings where temperature cannot be precisely regulated. The presence of antibodies in this lateral flow test also suggests that the test may have a relatively short shelf life,[36] however this value has not yet been reported. Additionally, the lateral flow test is qualitative and does not have the capacity to quantify HbS concentration (unlike the paper-based test presented here)[37] and therefore would not be useful for monitoring the effect of hydroxyurea and transfusion therapies. All of these potential issues notwithstanding, the lateral flow immunoassay could become a very useful confirmatory test, if the per-test total cost of the assay could be reduced below the cost of IEF, HE or HPLC performed currently as a standard of care.

There are two main limitations to the diagnostic capabilities of the paper-based test in its current form. The first limitation is that the test cannot accurately distinguish between patients with the HbSC form of sickle cell disease and those with sickle trait (HbAS) either based on visual scoring (**Fig 4A**) or the values of S-index (**Fig 5A**), because both HbSC and HbAS samples have similar levels of HbS (**Fig 4C**). We anticipate therefore that the utility of our paper-based test (as well as any other test relying solely on quantifying %HbS for the diagnosis of sickle cell disease) may be limited in Burkina Faso and surrounding countries of Central West Africa, where HbSC disease is most prevalent.[38] An important advantage of our assay, however, is that if digital images of the blood stains are acquired they can be analyzed in a number of ways to improve diagnostic accuracy. To demonstrate this capability, we devised the C-index that enabled us to distinguish between HbAS and HbSC within the group of heterozygous sickle cell trait carriers with sensitivity of 100% and specificity of 59% (**Fig 5B and 5C**). Such a high sensitivity, but relatively low specificity, means that C-index could be used to effectively screen out patients who do not have HbSC (negatives), but would necessitate confirmatory testing for patients who score positive to make a definitive diagnosis of HbSC. We also note that although hard threshold values were used for the purpose of evaluating sensitivity and specificity, a C-index close to the threshold values or the detection of any level of HbS in samples from patients with a high index of suspicion of SCA of some type could trigger more detailed testing by IEF or HPLC. Additionally, while automated image analysis to determine S- and C-index represents an additional layer of complexity to the performance of the test, it also offers significant advantages: the elimination of subjectivity in test scoring; the capacity for automated transmission and entry of results into a centralized database; and, when necessary, the quantitation of sickle hemoglobin levels[18] to guide therapeutic decision-making. Eventual adaptation of our automated test-scoring algorithm into software for camera-equipped and internet-capable portable devices (e.g., smart phones, tablets) could make such automated analysis feasible in remote settings and make it a significant asset in large-scale public health screening campaigns.

The second limitation of our current assay relates to its application in testing neonates and infants <12 month old. During gestation, 90–95% of all produced hemoglobin molecules are fetal hemoglobin (HbF), which is unaffected by the sickle mutation. Hemoglobin production switches to predominantly adult variants after 34–36 weeks of fetal development, and the fraction of HbF declines precipitously, reaching adult levels by age 8–12 months.[39, 40] At birth, the cord blood of normal newborns (HbAA) contains 14.3 ± 5.3% HbA, while blood of sickle cell trait carriers (HbAS) contains 9.5 ± 4.2% HbA and 6.5 ± 2.8% HbS, and blood of those with SCA (HbSS) contains 10.2 ± 3.9% HbS. The version of our paper-based test presented here is not capable of detecting these low levels of HbS present in blood of newborns and infants, limiting the usefulness of the test for the identification of SCA or sickle trait in this age group. To address this limitation, we are currently developing a newborn-specific version of our paper-based SCA test for rapid, highly sensitive detection of low levels of HbS in neonatal samples. Automated reading of neonatal and infant samples, as described above, could further increase the overall sensitivity of the test for HbS. While these improvements are unlikely to make a truly definitive diagnosis of newborns possible, a low-cost paper-based test sensitive to the low levels of HbS could facilitate rapid, inexpensive and efficient testing of large numbers of infants to screen out those negative for HbS, thus limiting expensive definitive testing (via IEF, HE or HPLC) and efforts to ensure follow-up care to the significantly smaller HbS-positive cohort.

In summary, we have refined and validated our previously developed paper-based assay for rapid, low-cost diagnostics of SCA at the point-of-care. We have demonstrated that the test can be used for the rapid and accurate diagnosis of sickle trait carriers and individuals with SCA (HbSS, HbSβ^+^-thalassemia) and, to a more limited extent, individuals with the HbSC form of sickle cell disease. We have also demonstrated that the test can be performed by novice healthcare workers in a resource-limited clinical setting. This simple, rapid and low-cost diagnostic test represents a major step towards enabling universal screening of children and adults for SCA in sub-Saharan Africa and other resource-limited settings.

## Supporting Information

S1 Fig Dependence of the blood stain pattern on the HbS content (%HbS) in the sample for three different formulations of the Hb solubility buffer containing 5, 30 or 100 g/L of sodium hydrosulfite ([Na_2_S_2_O_4_]). HbAA and HbSS blood samples (matched for ABO-Rh blood type and hemoglobin concentration) were mixed to artificially create samples with %HbS ranging from 0 to 75%.
(TIF)

S2 Fig Dependence of S-index on %HbS for three different formulations of the Hb solubility buffer containing 5, 30 or 100 g/L of sodium hydrosulfite. Four HbSS samples of known %HbS ((♦) HbS = 83.7%, [Hb] = 7.9 g/dL; (▲) HbS = 90.0%, [Hb] = 8.2 g/dL; (●) HbS = 88.4%, [Hb] = 9.1 g/dL; (■) HbS = 74.9%, [Hb] = 10.5 g/dL) were diluted with HbAA blood (matched for blood type and [Hb]) in ratios of 10:0, 9:1, 8:2, 7:3, 6:4, 5:5, 4:6, 3:7, 2:8, 1:9 and 0:10 (by volume) to create a range of reconstituted blood samples of known %HbS. The S-index for each reconstituted blood sample was measured using the three formulations of the Hb solubility buffer. The slope of linear fit was 12.1 for [Na_2_S_2_O_4_] = 30 g/L, in comparison to 6.8 HbS for [Na_2_S_2_O_4_] = 5 g/L and 15.9 HbS for [Na_2_S_2_O_4_] = 100 g/L. The standard deviation of the difference between the calculated and true %HbS was 6.4%HbS for [Na_2_S_2_O_4_] = 30 g/L, in comparison to 8.4%HbS for [Na_2_S_2_O_4_] = 5 g/L and 6.6%HbS for [Na_2_S_2_O_4_] = 100 g/L.
(TIF)

S3 Fig Variation of S-index due to imperfect metering of liquid volumes. Data shown as mean ± standard deviation (n = 5 blood stains per sample). (**a**) S-index measured for blood mixed with Hb solubility buffer at 1:8, 1:9, 1:10, 1:11 and 1:12 ratios by volume (droplet volume 20 μL). (**b**) S-index measured for 16 μL, 18 μL, 20 μL, 22 μL and 24 μL droplets of the mixture of blood and Hb solubility buffer (1:10 ratio, by volume).
(TIF)

S4 Fig Effect of delay in reading time and storage of blood samples. (**a, b**) Short-term stability of paper-based SCA assay measurements was evaluated by scanning the sheets of paper containing the blood stains repeatedly over (**a**) 1 hour and (**b**) 24 hours after the samples were deposited on paper (HbSS (■), HbAS (▲) and HbAA (●)). The shaded area in (**b**) outlines the data shown in (**a**). (**c**) S-index was measured daily for blood samples stored over a one week storage period. The error bars represent one standard deviation from the average of SCD assay measurements performed by 4 different technicians.
(TIF)

S5 Fig Supplementary Figure S5. Sample collection and classification flowchart for the Angola site.
(TIF)

S1 Methods Description of methods used to produce data for supplementary figures. (DOCX)

S1 Minimal Dataset The minimal data set underlying the findings reported in our study. (XLSX)

S1 Table Shows a breakdown of the costs of the paper-based test components. (DOCX)
