# Supplementary figures and images for: Validation of a Low-Cost Paper-Based Screening Test for Sickle Cell Anemia
Source: PLoS One. 2016 Jan 6;11(1):e0144901. doi: 10.1371/journal.pone.0144901 (PMC4703210; doi:10.1371/journal.pone.0144901)

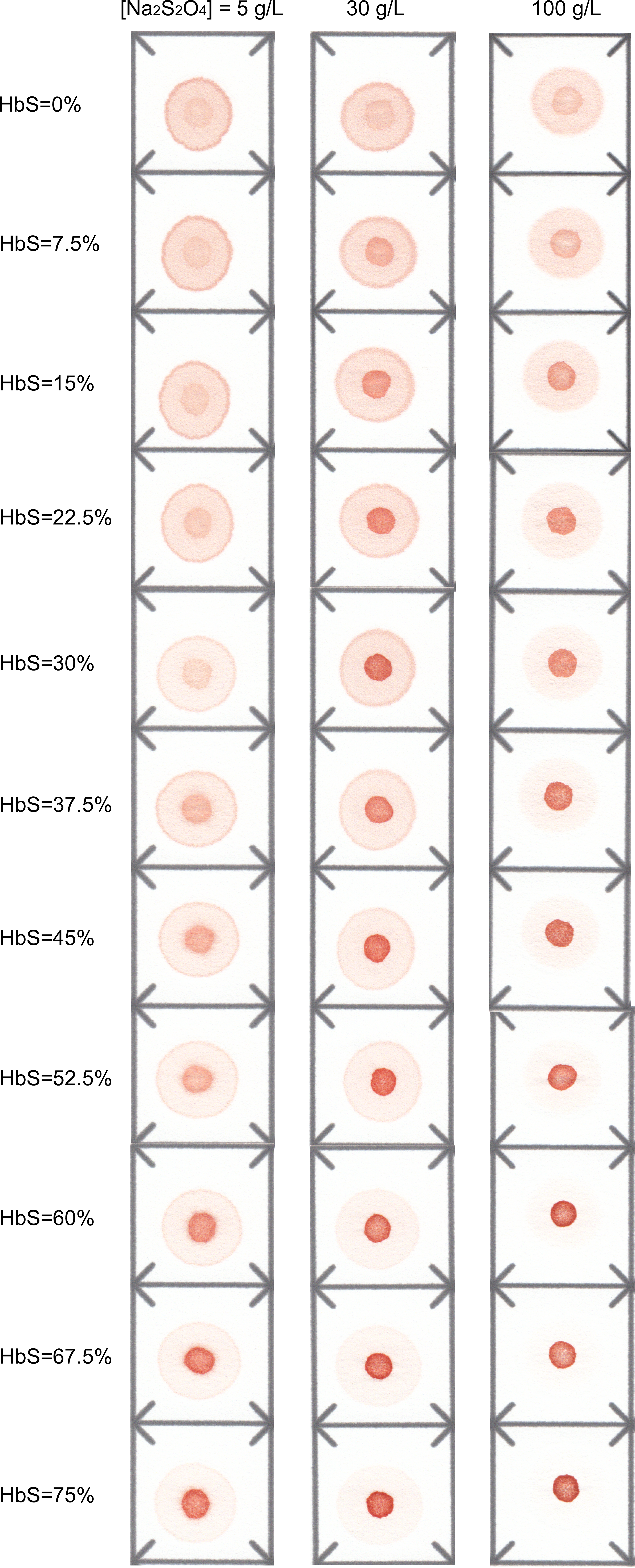

Supplement: S1 Fig — HbAA and HbSS blood samples (matched for ABO-Rh blood type and hemoglobin concentration) were mixed to artificially create samples with %HbS ranging from 0 to 75%. (TIF) [file pone.0144901.s001.tif]

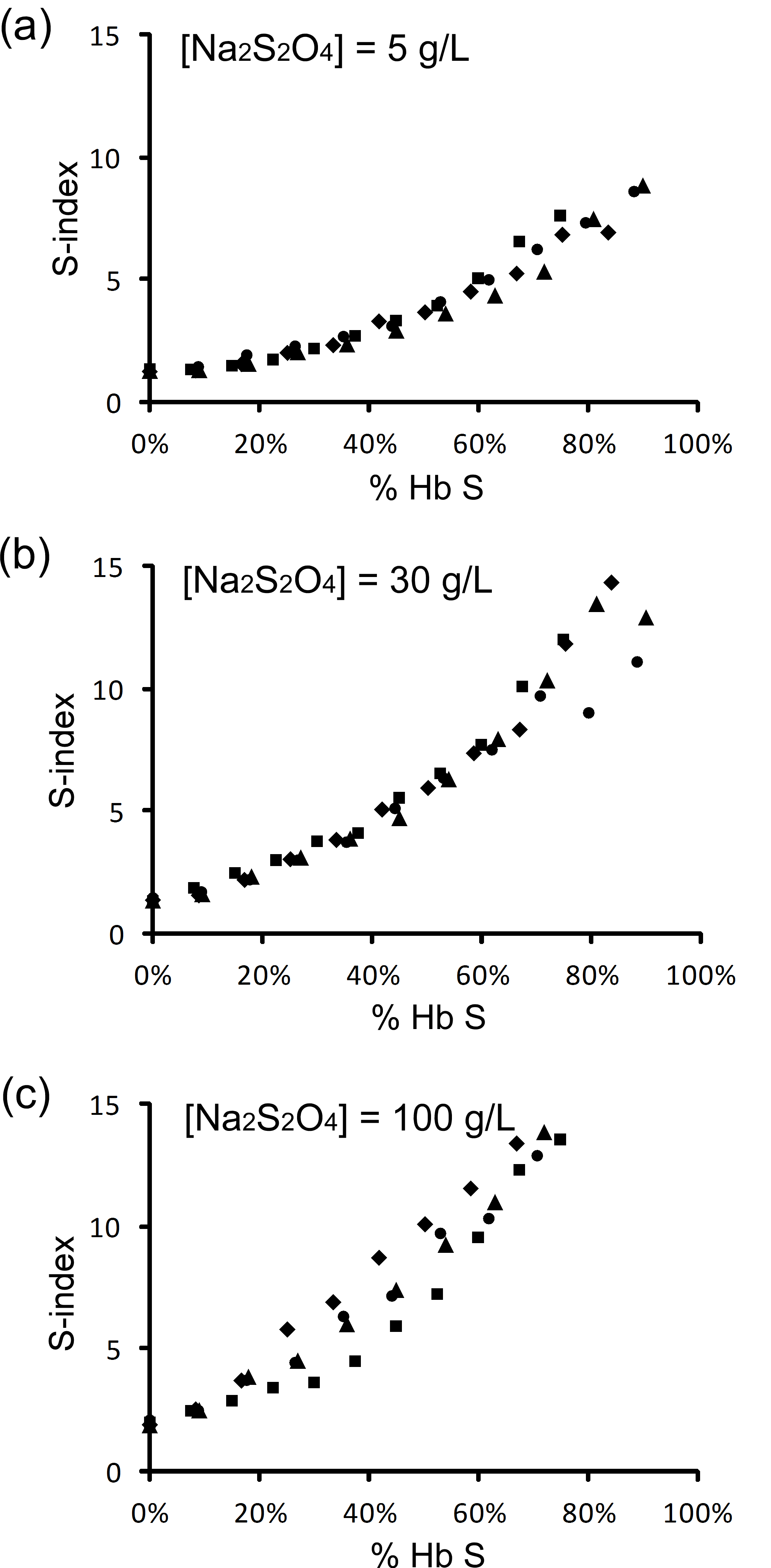

Supplement: S2 Fig — Four HbSS samples of known %HbS ((♦) HbS = 83.7%, [Hb] = 7.9 g/dL; (▲) HbS = 90.0%, [Hb] = 8.2 g/dL; (●) HbS = 88.4%, [Hb] = 9.1 g/dL; (■) HbS = 74.9%, [Hb] = 10.5 g/dL) were diluted with HbAA blood (matched for blood type and [Hb]) in ratios of 10:0, 9:1, 8:2, 7:3, 6:4, 5:5, 4:6, 3:7, 2:8, 1:9 and 0:10 (by volume) to create a range of reconstituted blood samples of known %HbS. The S-index for each reconstituted blood sample was measured using the three formulations of the Hb solubility buffer. The slope of linear fit was 12.1 for [Na2S2O4] = 30 g/L, in comparison to 6.8 HbS for [Na2S2O4] = 5 g/L and 15.9 HbS for [Na2S2O4] = 100 g/L. The standard deviation of the difference between the calculated and true %HbS was 6.4%HbS for [Na2S2O4] = 30 g/L, in comparison to 8.4%HbS for [Na2S2O4] = 5 g/L and 6.6%HbS for [Na2S2O4] = 100 g/L. (TIF) [file pone.0144901.s002.tif]

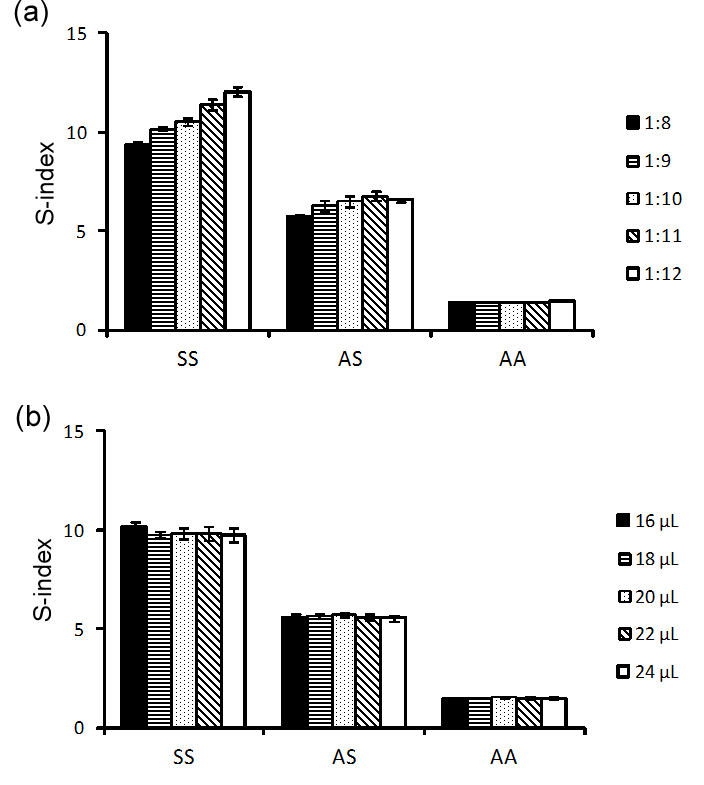

Supplement: S3 Fig — Data shown as mean ± standard deviation (n = 5 blood stains per sample). (a) S-index measured for blood mixed with Hb solubility buffer at 1:8, 1:9, 1:10, 1:11 and 1:12 ratios by volume (droplet volume 20 μL). (b) S-index measured for 16 μL, 18 μL, 20 μL, 22 μL and 24 μL droplets of the mixture of blood and Hb solubility buffer (1:10 ratio, by volume). (TIF) [file pone.0144901.s003.tif]

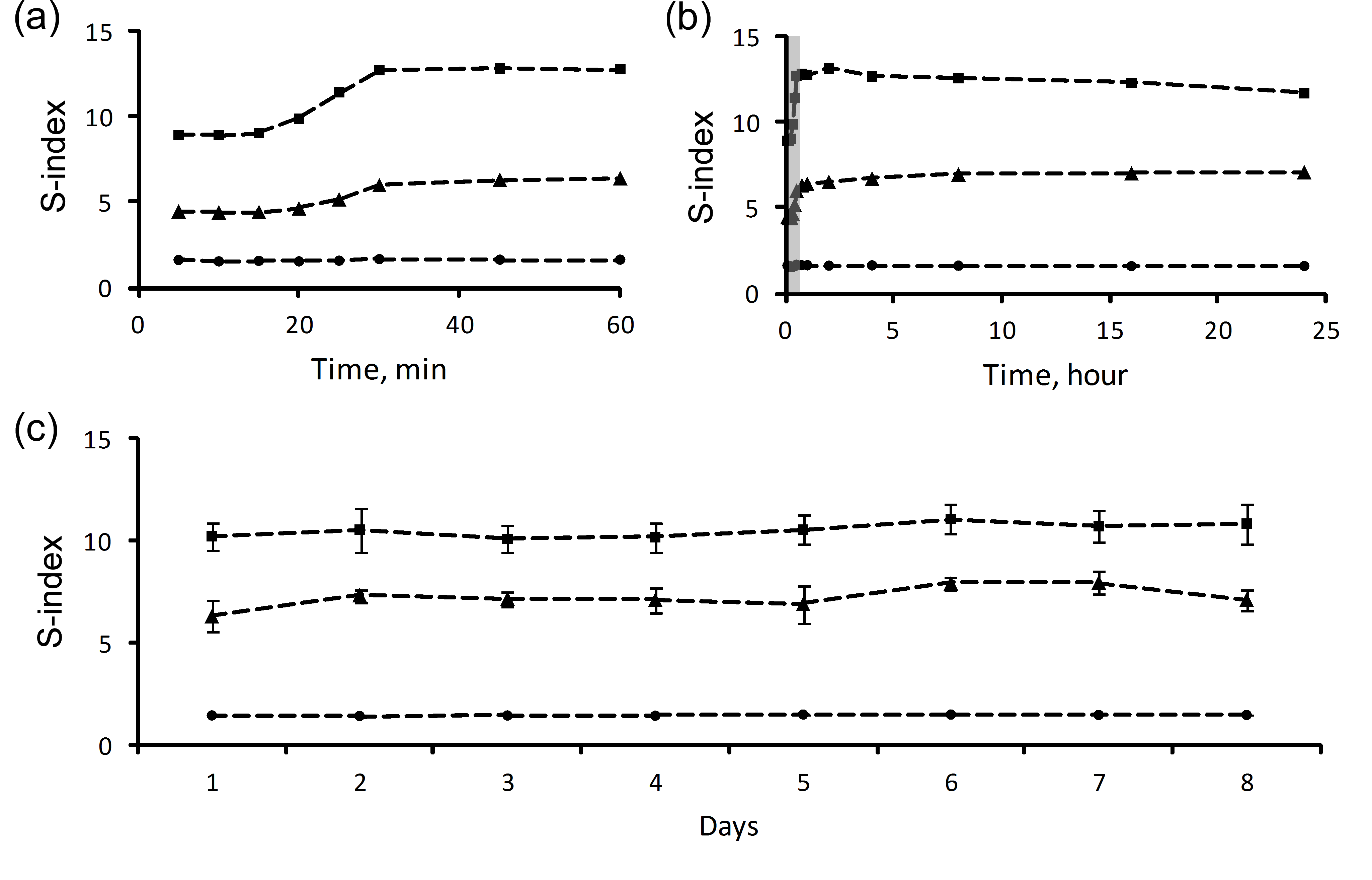

Supplement: S4 Fig — (a, b) Short-term stability of paper-based SCA assay measurements was evaluated by scanning the sheets of paper containing the blood stains repeatedly over (a) 1 hour and (b) 24 hours after the samples were deposited on paper (HbSS (■), HbAS (▲) and HbAA (●)). The shaded area in (b) outlines the data shown in (a). (c) S-index was measured daily for blood samples stored over a one week storage period. The error bars represent one standard deviation from the average of SCD assay measurements performed by 4 different technicians. (TIF) [file pone.0144901.s004.tif]

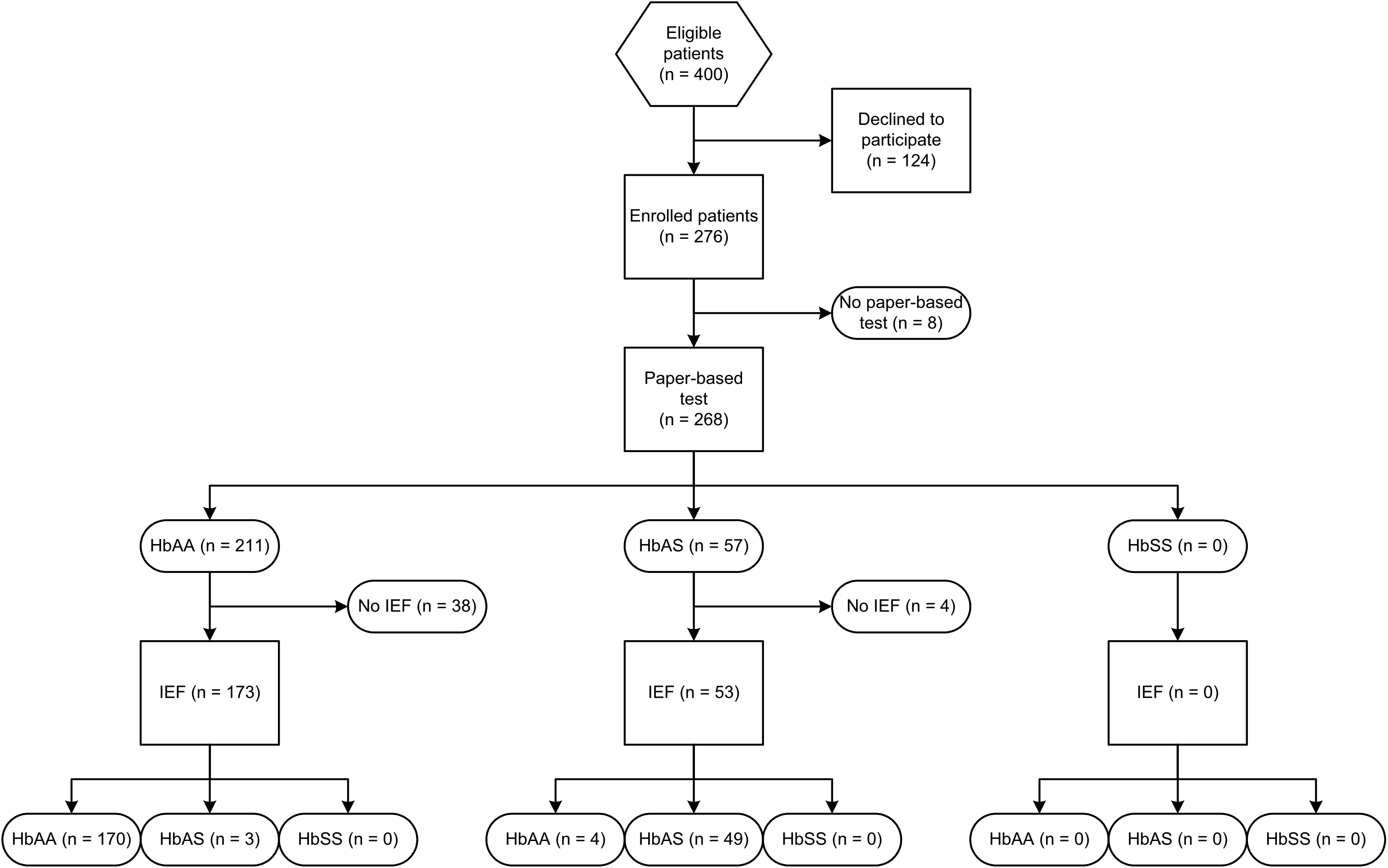

Supplement: S5 Fig — Sample collection and classification flowchart for the Angola site. (TIF) [file pone.0144901.s005.tif]
