# Supplementary material for: Validation of a Low-Cost Paper-Based Screening Test for Sickle Cell Anemia
Source: PLoS One. 2016 Jan 6;11(1):e0144901. doi: 10.1371/journal.pone.0144901 (PMC4703210; doi:10.1371/journal.pone.0144901)
Supplement: S1 Methods — (DOCX) [file pone.0144901.s006.docx]

**Supporting Information**

**Methods**

*Optimization of sodium hydrosulfite concentration*

In our previous work we used commercially-available SickleDex^TM^ as the hemoglobin solubility buffer. Here we reformulated the hemoglobin solubility buffer to improve reproducibility of our measurements and to increase the sensitivity of our paper-based SCA assay. Droplets with different %HbS produced blood stains with different color intensities of the center spot and the peripheral ring that were distinguishable visually (**S1 Fig**). We found that the concentration of sodium hydrosulfite, [Na_2_S_2_O_4_], had a strong effect on the performance of our assay. We used a series of reconstituted blood samples with artificially adjusted %HbS to determine optimal [Na_2_S_2_O_4_] for use in our assay. To prepare these samples, we collected several pairs of HbSS and HbAA blood samples (n = 4) matched for ABO-Rh blood type, adjusted the hemoglobin concentration ([Hb]) of HbAA samples to match that of the corresponding HbSS samples, and mixed these matched HbSS and HbAA samples at appropriate ratios to create a series of reconstituted samples with varying %HbS for each pair. Of the three [Na_2_S_2_O_4_] tested (5, 30 and 100 g/L), the dependence of S-index on %HbS for [Na_2_S_2_O_4_] = 30 g/L had a linear fit with a large slope and did not vary significantly between blood samples with different [Hb] (**S2 Fig**). We therefore used a Na_2_S_2_O_4_ concentration of 30 g/L to prepare the hemoglobin solubility buffer for all further experiments.

*Effect of variation in mixing ratio and droplet volume*

We measured the effect of variation in the blood / hemoglobin solubility buffer mixing ratio on the measurement of S-index for HbSS, HbAS and HbAA blood samples. We found that a ± 20% variation in the sample mixture ratio (1:8, 1:9, 1:10, 1:11, 1:12 by volume) resulted in S-index standard deviation of ≤ 1.06 (coefficient of variation, CV = 9.9%) for HbSS samples, ≤ 0.42 (CV = 6.6%) for HbAS samples, and ≤ 0.01 (CV = 0.9%) for HbAA samples (**S3a Fig**). We also measured the dependence of the S-index measurement on the volume of the sample droplet (a 1:10 mixture of blood and hemoglobin solubility buffer by volume) for HbSS, HbAS and HbAA blood samples. A ± 20% variation in the volume of the sample droplet (16, 18, 20, 22, 24 μL) resulted in S-index standard deviation of ≤ 0.15 (CV = 1.6%) for HbSS samples, ≤ 0.08 (CV = 1.4%) for HbAS samples, and ≤ 0.01 (CV = 0.8%) for HbAA samples (**S3b Fig**). The test was relatively insensitive to small variations in preparation of the blood lysate and spotting of the lysate onto the paper substrate.

*Stability of the paper-based assay*

We further tested stability of the paper-based SCA assay measurements with respect to delayed readout by scanning the sheets of paper containing the blood stains repeatedly over a period of 24 hours from the initial deposition of sample droplet on paper. The measurement of S-index showed a coefficient of variation (CV) of 3.1–15.6% over six scans performed within first 30 min (**S4a Fig**), and a CV of 1.6–5.8% for eight scans performed in the following 24 hours (**S4b Fig**). Finally, we tested variation in the S-index measurement for blood samples (HbAA, HbAS, HbSS) stored for up to a week before analysis. The S-index was 10.54 ± 0.35 (CV = 3.3%) for HbSS samples, 7.25 ± 0.54 (CV = 7.4%) for HbAS samples and 1.48 ± 0.03 (CV = 1.7%) for HbAA samples (**S4c Fig**). The test was relatively insensitive to small variations in test readout time (up to 24 hours after the stain has developed completely) and storage of the stain in paper for prolonged periods prior to digitization. It is somewhat sensitive, however, to test readout time within the 30 minute period before the stain has developed completely.

*Sample collection and classification*

**S5 Fig** shows a flowchart of sample collection and classification at the Angola site.
