# Supplementary material for: Validation of a Low-Cost Paper-Based Screening Test for Sickle Cell Anemia
Source: PLoS One. 2016 Jan 6;11(1):e0144901. doi: 10.1371/journal.pone.0144901 (PMC4703210; doi:10.1371/journal.pone.0144901)
Supplement: S1 Table — (DOCX) [file pone.0144901.s008.docx]

|  | **Cost per test (USD)** |
| --- | --- |
| **Test-specific components:** |  |
| Potassium phosphate monobasic | $0.0058 |
| Potassium phosphate dibasic | $0.0059 |
| Saponin (from quillaja bark) | $0.0053 |
| Sodium hydrosulfite | $0.0009 |
| Chromatography paper | $0.0522 |
| Water | $0.0001 |
| **Subtotal:** | **$0.0700** |
|  |  |
| **Blood collection components:** |  |
| Exam gloves | $0.1209 |
| Alcohol swab | $0.0296 |
| Single use lancet | $0.0375 |
| K_2_EDTA coated collection tube | $0.4056 |
| Bandage | $0.0303 |
| Pipette tips | $0.0541 |
| Tube | $0.0248 |
| **Subtotal:** | **$0.7029** |
|  |  |
| **Total:** | **$0.7730** |

**S1 Table** shows a breakdown of the costs of the paper-based test components.
